# 1278. Testing the Synergistic Effect of Ceftazidime-Avibactam Plus Aztreonam on Metallobetalactamase-Producing Non-Fermenting Gram-negative Bacteria

**DOI:** 10.1093/ofid/ofab466.1470

**Published:** 2021-12-04

**Authors:** Elena M Crouch, Sheila Johnson, Jason Bennett

**Affiliations:** 1 Uniformed Services University, CHEVY CHASE, Maryland; 2 Walter Reed Army Institute of Research, Silver Spring, Maryland

## Abstract

**Background:**

Metallo-betalactamases (MBL) are rapidly becoming a more widespread form of antimicrobial resistance. MBL are class B betalactamases that use zinc rather than serine in their active site and are only inactivated by monobactams, such as aztreonam. Unfortunately, most MBL-producing organisms also produce aztreonam-inactivating beta-lactamases. Synergy between ceftazidime-avibactam and aztreonam is well documented for MBL-producing Enterobacteriaceae but has not been tested extensively in non-fermenting Gram-negative bacteria. This study evaluates the susceptibilities of non-fermenting Gram-negative bacteria via E-test to this combination in vitro, in order to provide support for use to treat infections from these organisms.

**Methods:**

The antibiotic combination ceftazidime-avibactam+aztreonam was tested against a total of 33 isolates, including MBL-producing *Pseudomonas aeruginosa*, *Pseudomonas putida*, and the intrinsically aztreonam resistant *Acinetobacter baumanii* using the E-test method. MBL-producing Enterobacteriaceae were included as positive controls. All isolates were also tested against ceftazidime alone, aztreonam alone, and ceftazidime-avibactam. Bacterial isolates were procured from the Multidrug-resistant organism Repository & Surveillance Network at the Walter Reed Army Institute of Research. Antimicrobial resistance genes were previously identified by whole genome sequencing

**Results:**

Of 13 *Pseudomonas* spp. isolates tested, 9 were resistant, 3 were intermediate, and 1 was susceptible to aztreonam. Synergistic testing of ceftazidime-avibactam+aztreonam reduced the MIC of 4 *Pseudomonas* isolates by 1-2 doubling dilutions. While *Acinetobacter* spp. are usually considered intrinsically resistant to aztreonam, synergistic testing of ceftazidime-avibactam+aztreonam reduced the MIC of all 12 isolates tested by 1 to 3 doubling dilutions.

**Conclusion:**

The ability of ceftazidime-avibactam+aztreonam to reduce the MICs of *Acinetobacter baumanii* and MBL-producing *Pseudomonas aeruginosa* is a potentially promising therapeutic option when faced with growing antimicrobial resistance.

**Disclosures:**

**All Authors**: No reported disclosures

